# Handling method for GPS outages based on PSO-LSTM and fading adaptive Kalman filtering

**DOI:** 10.1038/s41598-025-95716-1

**Published:** 2025-04-07

**Authors:** Xiaoming Li, Xianchen Wang, Can Pei

**Affiliations:** 1 College of Surveying and Geo-Informatics, Tongji University, Shanghai, 200092 P. R. China; 2 School of Electronics and Communication Engineering, Shenzhen Polytechnic University, Shenzhen, 518115 P. R. China; 3 Internet of Things Research Institute, Shenzhen Polytechnic University, Shenzhen, 518115 P. R. China

**Keywords:** GPS/INS, Particle swarm optimization, LSTM, Fading adaptive filter, Computational science, Computer science, Engineering

## Abstract

To mitigate the degradation in GPS/INS integrated navigation performance during GPS signal outages, a PSO-optimized LSTM method is proposed to predict the pseudo position. The PSO algorithm is utilized to optimize two hyperparameters, neuron count and learning rate, which are essential to improve the training efficiency and prediction accuracy in the LSTM model. Considering that the predicted pseudo-position may contain outliers or accumulated errors, a robust algorithm is employed to mitigate its impact on correcting INS errors. Therefore, a Fading Adaptive Kalman Filter is introduced, which incorporates a dynamic fading factor to adaptively adjust the observation noise covariance matrix. This mitigates the impact of observation anomalies, further refining the filtering process. Experimental results demonstrate that the proposed PSO-LSTM method effectively reduces positional errors associated with inertial navigation during GPS outages and enhances the reliability of positioning. Compared to the conventional Extended Kalman Filter (EKF), the Fading adaptive EKF further improves three-dimensional positioning accuracy by up to 23.6%, 18.3%, and 22.7%, respectively.

## Introduction

Multi-sensor integrated navigation technology is widely used in navigation and positioning applications such as aerospace, autonomous driving, and railway inspection, with the combination of GNSS (Global Navigation Satellite System, GNSS) and INS (Inertial Navigation System, INS) being the most common^1–3^. Both GNSS and INS are considered passive positioning methods. GNSS can provide real-time high-precision positioning globally, but its drawback is that the signal is easily affected by building obstructions or spoofing, which can degrade positioning performance^4–6^. Inertial sensors are not affected by environmental conditions or weather and have a high sampling rate, enabling autonomous dead reckoning. However, they are susceptible to noise from accelerometers and gyroscopes, causing the errors in position and attitude estimation to rapidly diverge over time^7^. The integration of GNSS and INS can compensate for the shortcomings of using a single sensor in certain application scenarios. However, prolonged GNSS signal blockage can still make the integrated navigation system unreliable. Therefore, many scholars have conducted research on high-precision seamless positioning based on GNSS/INS integration in complex environments.

GNSS signals being in partially obstructed or obstructed areas generally refers to having fewer than four visible satellites, making standard single point positioning and precise point positioning infeasible. In this situation, introducing sensors such as magnetometers, wheel odometers, cameras, and LiDAR (Light Detection And Ranging, LiDAR) for auxiliary positioning is a feasible solution^8–11^. However, similar to inertial sensors, devices like odometer and cameras are prone to error accumulation and therefore rely on accurate external information to correct these inaccuracies. Magnetometers are susceptible to interference from metal objects and the Earth’s magnetic field. If a signal-obstructed area also experiences electromagnetic interference, positioning performance can degrade significantly. Due to its wide scanning range, long measurement distance, and high accuracy, LiDAR sensors have been widely used in integrated navigation applications. However, they are not the optimal choice for navigation and positioning scenarios that require low cost. In addition, altimeters and Doppler velocity sensors can also serve as supplements in a GNSS/INS multi-sensor integration. However, this can still lead to a relatively high system failure rate^12,13^, and data fusion between sensors may introduce additional errors. These include lever arm errors, time synchronization errors, initial alignment errors, and coordinate transformation errors, all of which can result in poor system robustness. Consequently, increasing research efforts are dedicated to advancing observation models and optimal estimation theories for GNSS/INS integration.

When GNSS signals are available, the most prevalent approach is the loosely coupled GNSS/INS integration, which utilizes position and velocity observations. Due to the highly nonlinear nature of the inertial navigation state equations, the EKF (Extended Kalman Filter, EKF) is typically employed for optimal estimation of the state errors from inertial sensors^14^. When calculating the Jacobian matrix of the state equations in the EKF, neglecting the higher-order terms of the Taylor expansion may lead to divergence. To improve this issue, the UKF (Unscented Kalman Filter, UKF) based on the UT transformation and the CKF (Cubature Kalman filter, CKF) utilizing the third-order spherical radial cubature criterion have been proposed. These approaches avoid linearization when tackling the optimal estimation problems of highly nonlinear systems^15–17^. However, both methods approximate the distribution of the state through sampled points, leading to an increase in computational complexity. In addition, PF (Particle Filtering, PF) and FKF (Federated Kalman Filtering, FKF) have also been introduced for optimal estimation of state parameters to enhance positioning accuracy^18,19^. However, these methods are not suitable for applications with stringent real-time requirements. Moreover, due to sensor limitations or changes in the operating environment, outliers may appear in the measurements. Standard nonlinear filtering estimators lack robust characteristics to handle such anomalies effectively. Therefore, adaptive robust Kalman filtering methods have been proposed to mitigate the impact of outliers on state estimation during measurement updates, such as IGG III and fading adaptive factors^20–22^. Two other commonly used GNSS/INS integration methods in partially obstructed environments are tight coupling and deep coupling. Tight coupling fuses data based on pseudo-range and pseudo-range rate, enabling more reliable navigation and positioning under multipath effects or interference^23,24^. Compared to tight coupling, deep coupling integrates inertial sensor data directly at the signal processing level within the GNSS receiver. In the signal tracking loop of the GNSS receiver, INS data can assist in satellite signal acquisition and tracking, especially in high-dynamic or signal-blocked environments^25^. However, compared to loosely coupled systems, both methods have more complex observation models or higher hardware design challenges, which limit their use in low-cost integrated navigation applications.

When GNSS signals are completely unavailable, the aforementioned optimal estimation methods and integration approaches cannot be applied. To prevent rapid error divergence from relying solely on inertial sensors, research on predicting GNSS pseudo-observations using various machine learning methods has been extensively developed^26^. evaluated the performance of various artificial intelligence methods, including MLP (Multilayer Perceptron, MLP) neural networks, RBF (Radial Basis Function, RBF) neural networks, wavelet neural networks, SVR (Support Vector Regression, SVR), and Adaptive Neuro-Fuzzy Inference Systems, for position prediction during GPS signal outages. Their results demonstrated significant improvements in accuracy compared to traditional methods. However, MLP neural networks are prone to getting stuck in local optima and are sensitive to the selection of hyperparameters. Reference^27^ utilized CNN (convolutional neural networks, CNN) to extract features from accelerometer and gyroscope data, while employing Gated Recurrent Units for modeling the time series. This approach accurately predicted the motion state of the vehicle during GNSS signal interruptions, effectively correcting INS errors. However, this method has a high training complexity and is more suitable for processing spatial data, such as images. Additionally, the longer training times can reduce the real-time applicability of the solution. In^28^’s study, the performance of a hybrid algorithm based on Gated Recurrent Units and Adaptive Kalman Filtering was compared with Kalman Filtering assisted by Multilayer Perceptrons, LSTM (Long Short-Term Memory networks, LSTM), and Gated Recurrent Units for GNSS position prediction. Additionally, it was demonstrated that Adaptive Kalman Filtering plays a crucial role in maintaining continuous and high-precision positioning for integrated navigation systems during signal interruptions^23^. In addition to using Adaptive Kalman Filtering to suppress the impact of outliers on the predicted values, the reference^29^ employed motion constraint detection to correct anomalies, thereby improving the positioning accuracy of GNSS/INS during signal interruptions^30^ and ^31^. employed machine learning methods such as Support Vector Machines and Least Squares Support Vector Machines to improve vehicle navigation accuracy in urban canyon environments. Both two methods require storing all training data and support vectors, which introduces storage pressure and computational limitations in real-time applications. Additionally, hyperparameters significantly impact their predictive performance. To address the issues, parameter optimization can be achieved using techniques such as PSO (Particle Swarm Optimization, PSO) or GA (Genetic Algorithms, GA)^32–34^. In summary, GNSS/INS integrated navigation assisted by LSTM methods can better leverage historical navigation data without the need to establish a physical motion model. It is well-suited for processing data in highly nonlinear systems, offers real-time processing potential, and demonstrates superior predictive performance^35–38^.

The paper aims to propose a continuous and reliable integrated navigation solution for scenarios where GPS signals are obstructed and unavailable. The solution employs a Particle Swarm Optimization-based LSTM model to predict displacement increments of the carrier during GPS outages. The main improvement is to utilize a PSO-based method for LSTM hyperparameter tuning to obtain globally optimal parameters, enhancing LSTM training efficiency and predictive performance. Additionally, it incorporates fading adaptive filtering to perform anomaly detection on the predicted increments, suppressing the impact of accumulated errors on the integrated navigation solution and thereby enhancing the reliability of the proposed approach when GPS is unavailable. The second section of the paper will introduce the principles of the GPS/INS state and observation models, the PSO-optimized LSTM model and the fading adaptive extended Kalman filter. In the third section, the proposed methods will be tested and validated using measured data. Finally, the discussion and conclusions of the study will be presented.

## Methods

### GPS/INS loosely coupled state model and observation model

The state space model and observation model of the loosely coupled GPS/INS integration are established in the ‘North-East-Down’ coordinate system, with the differences in position and velocity between GPS SPP (Stand Point Positioning, SPP) and INS dead reckoning used as observational inputs. The discretized loosely coupled state and observation models can be represented as follows:

1 $$\:\left\{\begin{array}{c}{X}_{k,k-1}={\varnothing\:}_{k-1,k-1}{X}_{k-1,k-1}+{W}_{k-1,k-1}\\\:{Z}_{k}={H}_{k}{X}_{k,k-1}+{V}_{k}\end{array}\right.$$

where $\:{X}_{k,k-1}$ represents the 19 state parameters which includes position errors, velocity errors, attitude errors, gyro drifts, accelerometer biases, lever arm errors of the INS to the GPS antenna and time synchronization error. $\:{\varnothing\:}_{k-1,k-1}$ is the state transition matrix. $\:{Z}_{k}$ represents the difference in position between GPS and INS. $\:{H}_{k}$ is the observation matrix at epoch $\:k$. $\:{W}_{k-1,k-1}$ and $\:{V}_{k}$ represent the state noise vector and observed noise vector, which the corresponding covariance matrices can be written as $\:{Q}_{k-1,k-1}$ and $\:{R}_{k}$. The filter used here is the EKF, therefore, $\:{\varnothing\:}_{k-1,k-1}$ and $\:{H}_{k}$ matrices represent the Jacobian matrix of the INS state errors.

The detailed derivation of the coefficient matrices related to position, velocity, and attitude errors in the state transition matrix $\:{\varnothing\:}_{k-1,k-1}$ of the INS state equation can be found in^39^. When GPS is available, its output is typically used as the primary reference. However, when GPS is unavailable, data fusion between the two sensors cannot be performed. Therefore, a machine learning approach is proposed to predict pseudo position increments of the vehicle during GPS outages, which can then be fused with the position derived from the INS. The primary concept is to establish a mapping relationship between the outputs of the accelerometer and gyroscope and the position increments from GPS/INS integrated navigation when GPS signals are available. When the signals are completely blocked, the vehicle’s position increments are predicted based on the outputs of the inertial sensors. This information, along with the position data before the signal interruption, is used to compute the position during the interruption for measurement updates.

### PSO-based LSTM model training and forecasting

**Fig. 1:**
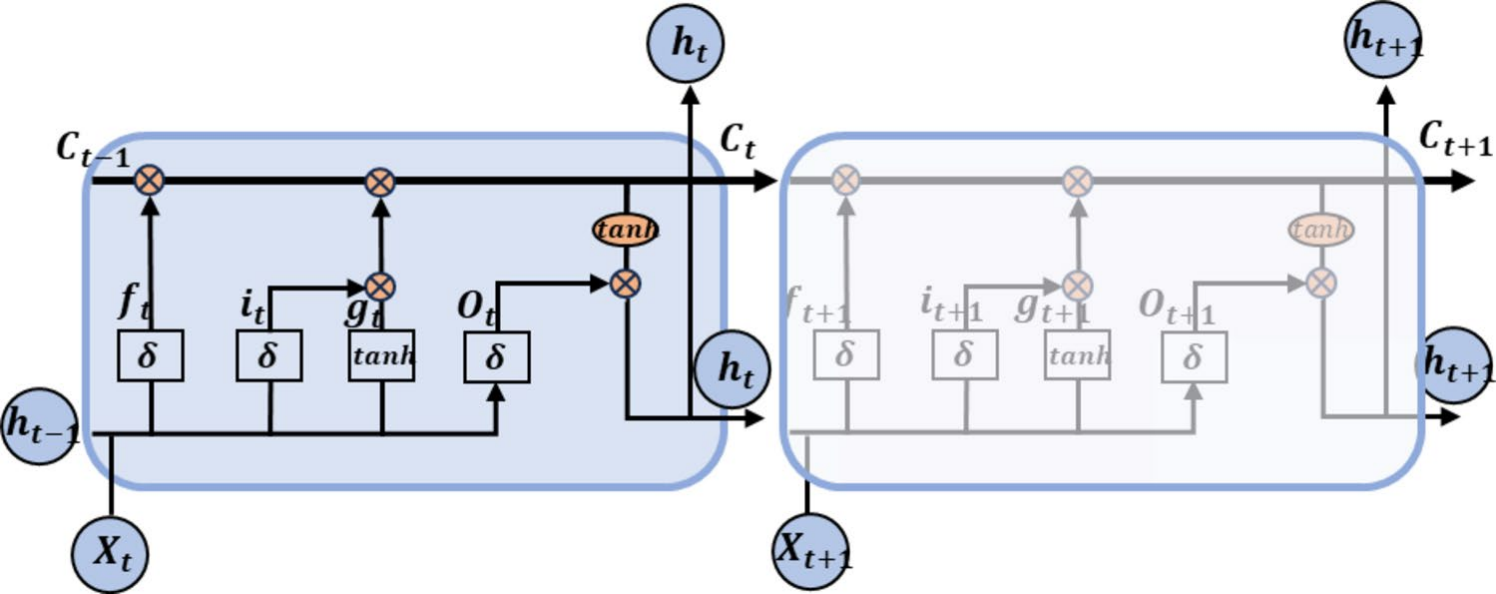
The LSTM network architecture.

The LSTM model features a straightforward structure with fewer parameters. During training, the LSTM network adjusts its weights using the backpropagation algorithm while minimizing the loss function through gradient descent. During prediction, the network utilizes its memory cells to retain crucial historical information, effectively capturing long-term dependencies in time series data. This capability makes LSTM particularly effective for processing sequential data. The LSTM network can be represented by memory cells connected at adjacent time steps, with the basic results illustrated in Fig. 1.

Each rectangular block in Fig. 1 represents a memory block, which consists of a forget gate $\:{f}_{t}$, an input gate $\:{i}_{t}$, an output gate $\:{O}_{t}$, and a state memory block $\:{C}_{t-1}$. $\:{g}_{t}$ is used to modify the updated value of $\:{C}_{t-1}$. $\:{X}_{t}$ and $\:{h}_{t-1}$ represent the input at the current time which passed through the forget gate to determine the part of the state memory block needs to be forgotten. The vector that needs to be retained in the state memory block is determined by the sigmoid function ($\:\delta\:$) of the input gate and the $\:tanh$ activation function. The final output $\:{h}_{t}$ is obtained by the updated state memory block $\:{C}_{t}$ and the output gate. The input and output steps can be expressed by the following equations.

2 $$\:{f}_{t}=\delta\:\left({W}_{f}\cdot\:\left[{h}_{t-1},{x}_{t}\right]+{b}_{f}\right)$$

3 $$\:{i}_{t}=\delta\:\left({W}_{i}\cdot\:\left[{h}_{t-1},{x}_{t}\right]+{b}_{i}\right)$$

4 $$\:{g}_{t}=tanh\left({W}_{g}\cdot\:\left[{h}_{t-1},{x}_{t}\right]+{b}_{g}\right)$$

5 $$\:{C}_{t}={f}_{t}\cdot\:{C}_{t-1}+{i}_{t}\cdot\:{g}_{t}$$

6 $$\:{O}_{t}=\delta\:\left({W}_{O}\cdot\:\left[{h}_{t-1},{x}_{t}\right]+{b}_{O}\right)$$

7 $$\:{h}_{t}={O}_{t}\cdot\:tanh\left({C}_{t}\right)$$

where $\:{W}_{f}$, $\:{W}_{i}$, $\:{W}_{O}$ and $\:{W}_{g}$ represent the weight of the forget gate, input gate, output gate and state memory block. $\:{b}_{f}$, $\:{b}_{i}$, $\:{b}_{O}$ and $\:{b}_{g}$ represent the deviation of the forget gate, input gate, output gate and state memory block.

In an LSTM network, the learning rate controls the step size for weight updates during gradient descent, and setting it too high or too low can lead to unstable training. The number of neurons in the hidden layer directly impacts the network’s representational capacity. Therefore, to avoid a decline in prediction performance caused by using empirical hyperparameters for model training, this paper innovatively proposes the method of a global parameter optimization to optimize the learning rate and the number of neurons in the LSTM model, aiming to achieve optimal training and prediction performance. Compared to genetic algorithms and others, the Particle Swarm Optimization algorithm demonstrates advantages in terms of convergence speed, adaptability, and computational complexity^40^. Therefore, in this study, PSO is applied to optimize these two hyperparameters before using the LSTM network to predict position and velocity increments during GPS outages, improving both the training efficiency and predictive performance of the model.

Assume that the number of neurons and the learning rate are represented by symbols $\:\alpha\:$ and $\:\epsilon\:$, respectively. The initial dimensionality of the population and the number of particles in each population are represented by $\:D$ and $\:N$. The initial position information $\:{x}_{i}$ and velocity information $\:{v}_{i}$ corresponding to the two parameters $\:\left(\alpha\:,\epsilon\:\:\right)$ of each particle are randomly obtained within a predefined range, where $\:i=\text{1,2},\dots\:,\:N$. During each iteration, particles update their velocity and position using the following equation,

8 $$\:\left\{\begin{array}{c}{v}_{i,d}^{k}=w{v}_{i,d}^{k-1}+{c}_{1}{r}_{1}\left({pbest}_{i,d}^{k}-{x}_{i,d}^{k-1}\right)+{c}_{2}{r}_{2}\left({gbest}_{d}^{k}-{x}_{i,d}^{k-1}\right)\\\:{x}_{i,d}^{k}={x}_{i,d}^{k-1}+{v}_{i,d}^{k-1}\end{array}\right.$$

where $\:{v}_{i,d}^{k}$ represents the $\:d$-th dimension velocity vector of particle $\:i$ at the $\:k$-th iteration ($\:d=1,\dots\:,D$). $\:{c}_{1},{c}_{2}$ represent the individual learning factor and the group learning factor, respectively. $\:{r}_{1},{r}_{2}$ represent random numbers within the interval [0, 1], increasing the randomness of the search. $\:{x}_{i,d}^{k-1}$ denotes the $\:d$-th dimension position vector of particle $\:i$ at the $\:k$-1-th iteration ($\:d=1,\dots\:,D$). It should be noted that both the position and velocity of the particles should be constrained within a certain range to avoid getting trapped in local optima or missing the global optimal solution. $\:w$ represents the inertia weight which used to adjust the search range of the solution space. $\:{pbest}_{i,d}^{k}$ represents the individual best solution of the $\:i$-th particle in the $\:d$-th dimension during the $\:k$-th iteration, while $\:{gbest}_{d}^{k}$ denotes the global best solution of the population in the $\:d$-th dimension during the $\:k$-th iteration.

To select the global optimal solution for the two hyperparameters $\:\left(\alpha\:,\epsilon\:\:\right)$ from the population, we calculate the fitness function value for each particle. The fitness function $\:{F}_{fitness}$ is constructed as follows

9 $$\:{F}_{fitness}=\sqrt{\raisebox{1ex}{$\sum\:_{j=1}^{Num}{\left({\stackrel{\sim}{y}}_{j}-{y}_{j}\right)}^{2}$}\!\left/\:\!\raisebox{-1ex}{$Num$}\right.}$$

where $\:Num$ represents the number of training samples. $\:{y}_{j}$ is the network’s training output. $\:{\stackrel{\sim}{y}}_{j}$ denotes the predicted output. Therefore, in each iteration, the optimal solution for the particles in the population is searched based on the calculated fitness function values.

### Anomaly detection and fading adaptive filtering

Considering that the GPS pseudo position predicted by the PSO-based LSTM network may contain outliers, relying solely on the prediction residuals may not be sufficient to fully detect anomalies in the observation model while filtering estimation, potentially leading to unreliable estimation results. Thereafter, a test statistic is constructed based on the prediction residual information to identify observation anomalies.

The test statistic follows a standard normal distribution and can be expressed as.

10 $$\frac{{{\Delta_{{k_i}}}}}{{{\delta _{{\Delta_{{k_i}}}}}}} \sim \rm N\left( {{\rm{0,1}}} \right)$$

where $\:{\varDelta\:}_{{k}_{i}}$ denotes the predicted residual and corresponding standard error is $\:{\delta\:}_{{\varDelta\:}_{{k}_{i}}}$, which can be calculated by Eqs. (11) and (12).

11 $$\:{\varDelta\:}_{{k}_{i}}={Z}_{k}-{H}_{k}{\stackrel{\sim}{X}}_{k,k-1}$$

12 $$\:{\delta\:}_{{\varDelta\:}_{{k}_{i}}}={\left({H}_{{k}_{i}}{P}_{{k}_{i},{k}_{i}-1}{H}_{{k}_{i}}^{T}+{\delta\:}_{i}^{2}\right)}^{\raisebox{1ex}{$1$}\!\left/\:\!\raisebox{-1ex}{$2$}\right.}$$

where $\:{\stackrel{\sim}{X}}_{k,k-1}$ is the one-step prediction value of the state. $\:{\delta\:}_{i}^{2}$ represents the diagonal elements of the observation noise covariance matrix. $\:{P}_{{k}_{i},{k}_{i}-1}$ denotes the one-step prediction value of the state covariance. $\:i$ is the row element of observation matrix at epoch $\:k$. Assuming a confidence level of $\:(1\:-\:\alpha\:)\%$, if the test statistic exceeds the threshold, it indicates the presence of observation anomalies. Thereby, a fading adaptive factor would be introduced to dynamically adjust the observation noise covariance matrix, mitigating the influence of anomalous observations.

As shown in Eq. (12), omitting the subscript $\:i$, the covariance of the prediction residuals $\:E\left({\varDelta\:}_{k}{\varDelta\:}_{k}^{T}\right)$ can be expressed as

13 $$\:E{\left({\varDelta\:}_{k}{\varDelta\:}_{k}^{T}\right)=H}_{k}{P}_{k,k-1}{H}_{k}^{T}+{R}_{k}$$

where $\:{R}_{k}$ is the observation noise variance matrix. The variance of the prediction residuals represents the ensemble average of the random sequence and can be replaced by the time average in the discretized equation. Thus, the Eq. (13) can be rewritten as.

14 $$\:{R}_{k}=\left(1-\frac{1}{k}\right){R}_{k-1}+\frac{1}{k}\left({\varDelta\:}_{k}{\varDelta\:}_{k}^{T}-{{H}_{k}P}_{k,k-1}{H}_{k}^{T}\right)$$

Observation noise is primarily caused by external factors, making it prone to significant changes. Fixed observation noise cannot effectively accommodate dynamic systems, and adaptive adjustments based on historical and current observation information are required. The fading adaptive factor can fully utilize both historical observation noise information and prediction residual information. Therefore, the fading factor $\:b\left(0<b<1\right)$ is introduced and adaptive factor is represented as

15 $$\:{\gamma\:}_{k}=\frac{{\gamma\:}_{k-1}}{{\gamma\:}_{k-1}+b}$$

The Eq. (14) can be rewritten as.

16 $$\:{R}_{k}=\left(1-{\gamma\:}_{k}\right){R}_{k-1}+{\gamma\:}_{k}\left({\varDelta\:}_{k}{\varDelta\:}_{k}^{T}-{{H}_{k}P}_{k,k-1}{H}_{k}^{T}\right)$$

**Fig. 2:**
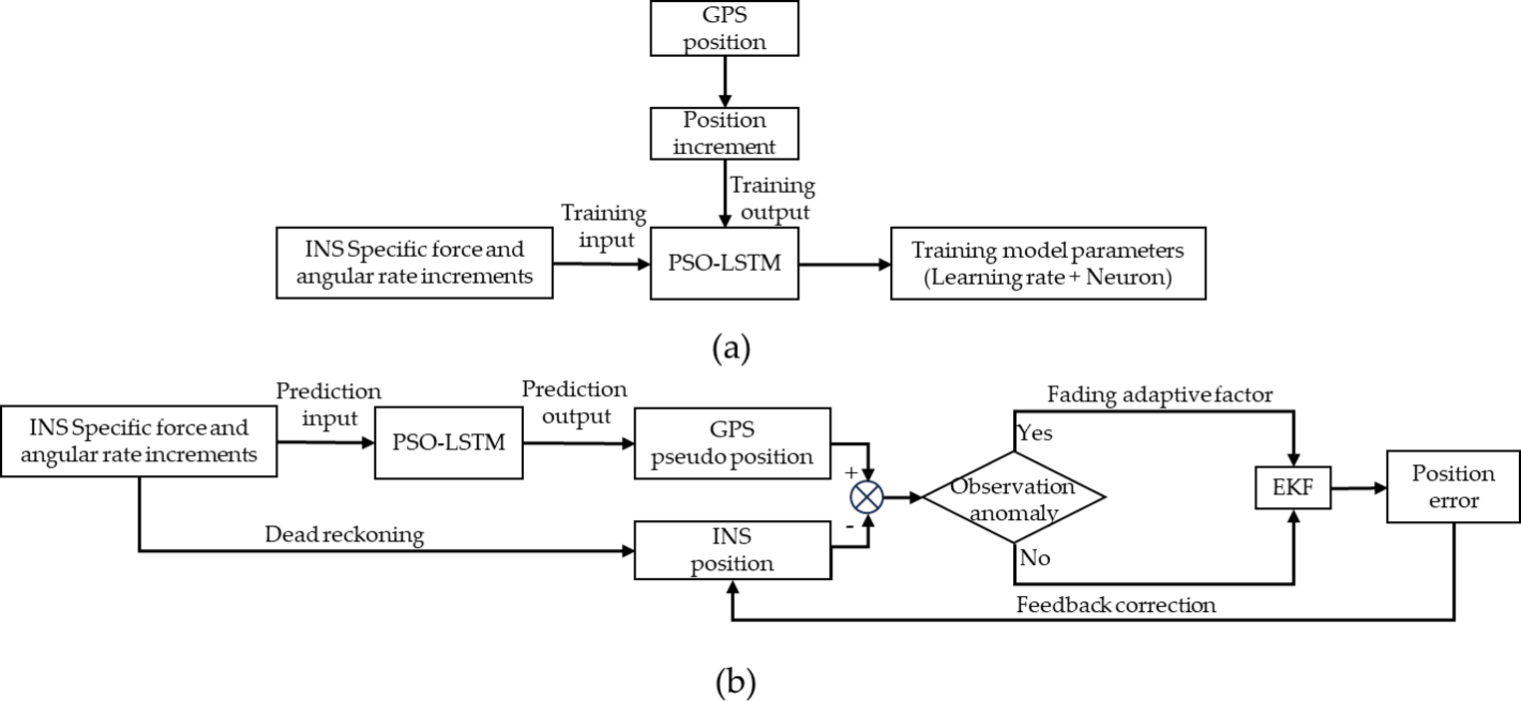
The GPS/INS adaptive integrated navigation method based on PSO-LSTM during GPS outages. (**a**) Training stage. (**b**) Prediction stage.

It can be seen that adaptive factor increases the contribution of current observation information to state estimation. The above constitutes the integrated navigation scheme proposed in this paper for scenarios where GPS signals are unavailable and the corresponding process for training and prediction stage is illustrated in Fig. 2. Extend Kalman Filter is utilized, with the state and measurement update steps outlined as follows.

State update,

17 $$\:\left\{\begin{array}{c}{\stackrel{\sim}{X}}_{k,k-1}={\varnothing\:}_{k-1,k-1}{X}_{k-1,k-1}\\\:{P}_{k,k-1}={\varnothing\:}_{k-1,k-1}{P}_{k-1,k-1}{\varnothing\:}_{k-1,k-1}^{T}+{Q}_{k-1,k-1}\end{array}\right.$$

Measurement update,

18 $$\:\left\{\begin{array}{c}{K}_{k}={P}_{k,k-1}{H}_{k}^{T}{\left({{H}_{k}P}_{k,k-1}{H}_{k}^{T}+{R}_{k}\right)}^{-1}\\\:{P}_{k}=\left(I-{K}_{k}{H}_{k}\right){P}_{k,k-1}\\\:{\stackrel{\sim}{X}}_{k}={\stackrel{\sim}{X}}_{k,k-1}+{K}_{k}\left({Z}_{k}-{H}_{k}{\stackrel{\sim}{X}}_{k,k-1}\right)\end{array}\right.$$

where $\:{K}_{k}$ represents the gain matrix, $\:{P}_{k}$ denotes the estimated state covariance matrix, and $\:{\stackrel{\sim}{X}}_{k}$ is the filtered estimation values.

## Experiment and results

### Experimental setup

**Fig. 3:**
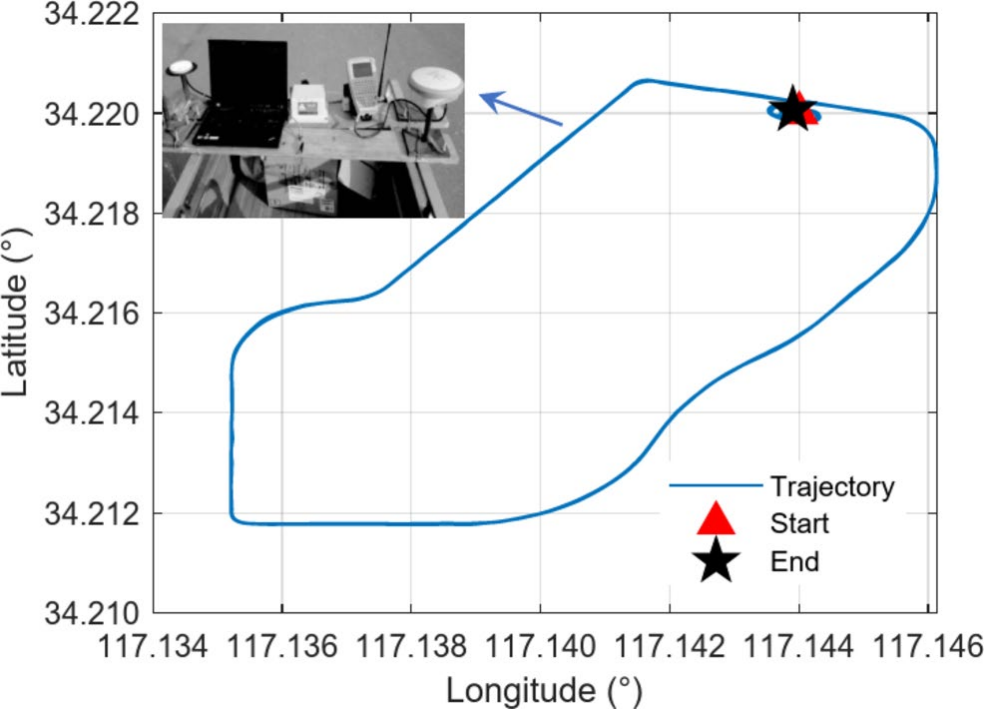
Trajectory of vehicles in the geodetic coordinate system.

The experimental setup for GPS and INS data acquisition consists of a Leica 1200 GPS receiver and a SPAN-CPT integrated navigation and positioning system. The GPS receiver is configured with a sampling rate of 10 Hz, while the IMU operates at a higher rate of 100 Hz. The data collection was conducted over a total duration of approximately 1810 s. The vehicle was driven along a diverse trajectory, designed to include straight segments, turns, and varying speeds. Differential positioning was achieved using a static base station collocated within the vicinity of the test area, providing reference positions with centimeter-level accuracy. For clarity, Fig. 3 illustrates the vehicle’s trajectory, including its start and end points, alongside the experimental equipment mounted on the test vehicle.

For IMU error modeling, the biases and random walk parameters of the accelerometer and gyroscope are detailed in Table 1. The INS was initialized with position errors of 1 m in the North and East directions, and 1.5 m in the Down direction. The initial velocity errors were set to 0.5 m/s in all three directions. Additionally, attitude errors were introduced with initial values of 1° in pitch and roll, and 3° in heading.

The GPS measurements were modeled with positioning error standard deviations of 0.5 m for the horizontal components and 1 m for the Down component, which align with typical performance metrics of the Leica 1200 receiver under differential GNSS mode. The extended Kalman filter utilized in this study was initialized using the aforementioned settings. Specifically, the initial state covariance matrix was constructed based on the INS position, velocity, and attitude errors, while the observation noise and state covariance matrix was derived from the GPS error standard deviations and IMU noise characteristics.

**Table 1:** Bias and random walk parameters of inertial sensor.

	Bias	Random walk
Accelerometer	$\:50mg$	$\:50\mu\:g/\sqrt{hour}$
Gyroscope	$\:{20}^{^\circ\:}/hour$	$\:{0.067}^{^\circ\:}/\sqrt{hour}$

**Fig. 4:**
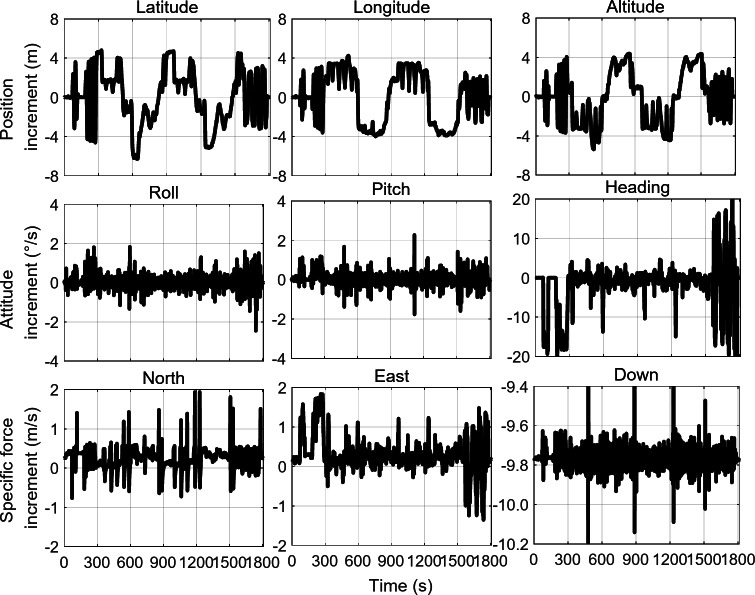
Position increments, attitude increments, and specific force increments during the vehicle’s motion.

The mechanization of inertial navigation calculates the velocity and displacement increments of the vehicle by performing integral operations on the outputs of the accelerometer and gyroscope. The specific force and angular rate outputs of inertial sensors exhibit a nonlinear relationship with displacement increments. Consequently, the vehicle’s motion characteristics, such as straight-line movement, turns, climbs, and accelerations, are also reflected in the sensor output information. Figure 4 shows the displacement increments in the latitude, longitude, and altitude directions, as well as the sensor output information for the vehicle moving along the trajectory displayed in Fig. 3. It can be observed that when the vehicle is making a turn, the heading rate exhibits significant changes, while during linear acceleration or deceleration, the specific force output shows noticeable variations. This indicates a correlation between changes in the vehicle’s motion state and variations in the raw output information from the sensors.

To validate that the proposed method can effectively predict vehicle displacement increments during GPS signal outages, a simulation was conducted where the GPS is available from 1 to 1200 s and unavailable from 1201 to 1810 s. Based on the available periods, the specific force and angular rate outputs from the inertial sensors serve as input for LSTM training, with the corresponding reference displacement increments as the training output. Firstly, a PSO algorithm is employed to iteratively optimize the number of neurons and learning rate parameters to obtain the global optimal values. To demonstrate the effectiveness and feasibility of the proposed method, MLP, GRU (Gated Recurrent Unit, GRU) and LSTM methods with empirically determined hyperparameters were used to train and predict position increments. Their results were compared with the PSO-LSTM scheme. The accuracy was evaluated using three metrics: RMSE (Root Mean Square Errors, RMSE), MAE (Mean Absolute Error, MAE), and R-squared. The parameter settings for each machine learning model are provided in Table 2.

**Table 2:** Parameters settings of LSTM, PSO, MLP and GRU.

LSTM	Input dimension	Output dimension	Optimizer	Max Epochs	Gradient Threshold	Neurons	Learning Rate
	6	3	Adam	50	1	50	0.01
PSO	Population Size	Dimensions	Max Iterations	Learning Factor 1	Learning Factor 2	Maximum Inertia Weight	Minimum Inertia Weight
	20	2	50	2	2	0.9	0.4
MLP	Goal Error	Max Epochs	Hidden Layer Size	Learning Rate	Min grad	-	-
	1.0E-03	100	20	0.01	1.0E-07	-	-
GRU	Goal Error	Max Epochs	Hidden Layer Size	Learning Rate	-	-	-
	1.0E-03	100	64	0.01	-	-	-

### Training and testing results

**Fig. 5:**
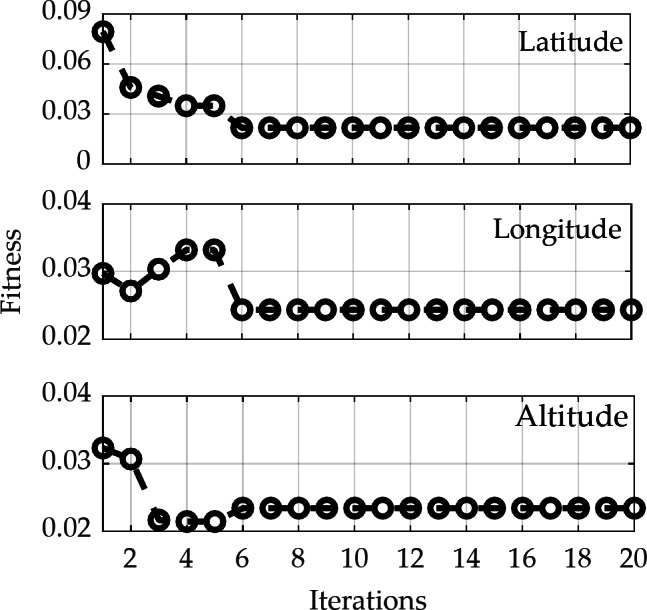
PSO parameter optimization fitness curve.

**Fig. 6:**
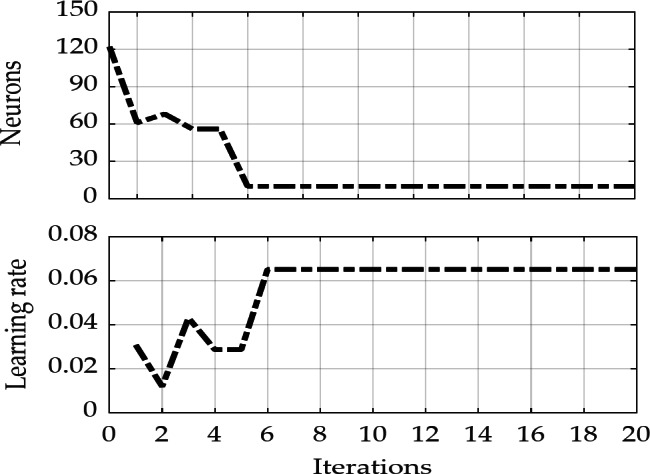
Optimization of the number of neurons and learning rate parameters in the LSTM model.

Figure 5 illustrates the iterative results of the fitness function values in the latitude, longitude, and altitude directions. It is evident that the LSTM training fitness values for all three directions converge after six iterations of PSO optimization. At this stage, the globally optimal values for the number of neurons and the learning rate are determined to be 10 and 0.0651, respectively, for training and predicting with the LSTM model. Figure 6 presents the optimization results of the two hyperparameters, the number of neurons and learning rate, during each PSO iteration for the LSTM model. This indicates that after six iterations, the global optimal positions and values for both hyperparameters have been identified. It also demonstrates that relying solely on empirical values to replace LSTM model hyperparameters does not ensure effective training and prediction performance.

**Fig. 7:**
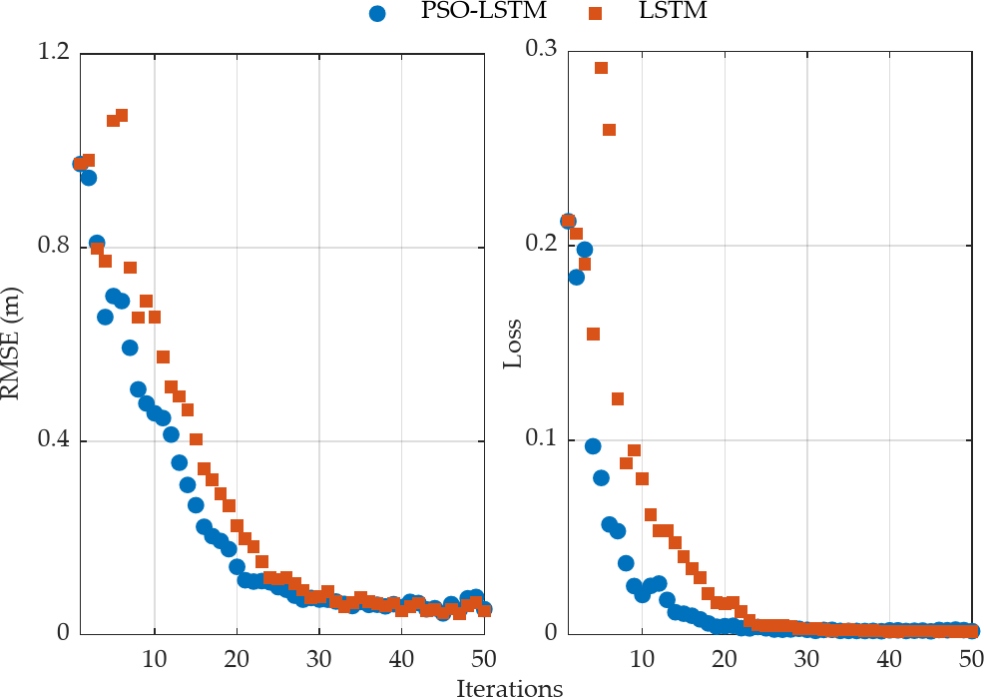
RMSE and loss function values of PSO-LSTM and LSTM models on training set data.

When training the LSTM model based on the optimized hyperparameters for the number of neurons and learning rate, it is important to set an appropriate number of iterations to ensure that the model has sufficient opportunities to learn data features while avoiding overfitting. Moreover, it can also reduce the training time. As shown in Fig. 7, the RMSE and Loss of the training for the PSO-LSTM model tends to stabilize as the number of iterations increases. After approximately 22 iterations, the RMS training error between adjacent iterations can be reduced to less than 1 mm. The loss function value approaches 0.01 after about 13 iterations. In contrast, when training the LSTM model using empirical model parameters directly, the number of iterations required to reach convergence for both the training error and loss function value exceeds that of the PSO-optimized LSTM model, undoubtedly affecting both the model’s performance and the training time. It is essential to employ a PSO algorithm for global hyperparameter optimization.

Regarding the time consumption for hyperparameter optimization, model training, and prediction using the proposed model, the average time for a single PSO fitness value calculation is approximately 17.21 s. Model training, based on the optimized parameters, number of neurons and learning rate, takes about 30.89 s, while the forecasting time is approximately 0.09 s. The computer used for data processing is configured with 16 GB of RAM and a 3.70 GHz CPU. The total time for the global optimization process of the LSTM model hyperparameters depends on the chosen population size and the maximum number of iterations, and the optimization can be offline calibrated using historical data. After optimization, the total time for online training of the LSTM model with approximately 12,000 samples is 30.89 s. If the sample size is reduced, this time will decrease further. The predicting time has negligible impact on the real-time operation of the GPS/INS system.

**Fig. 8:**
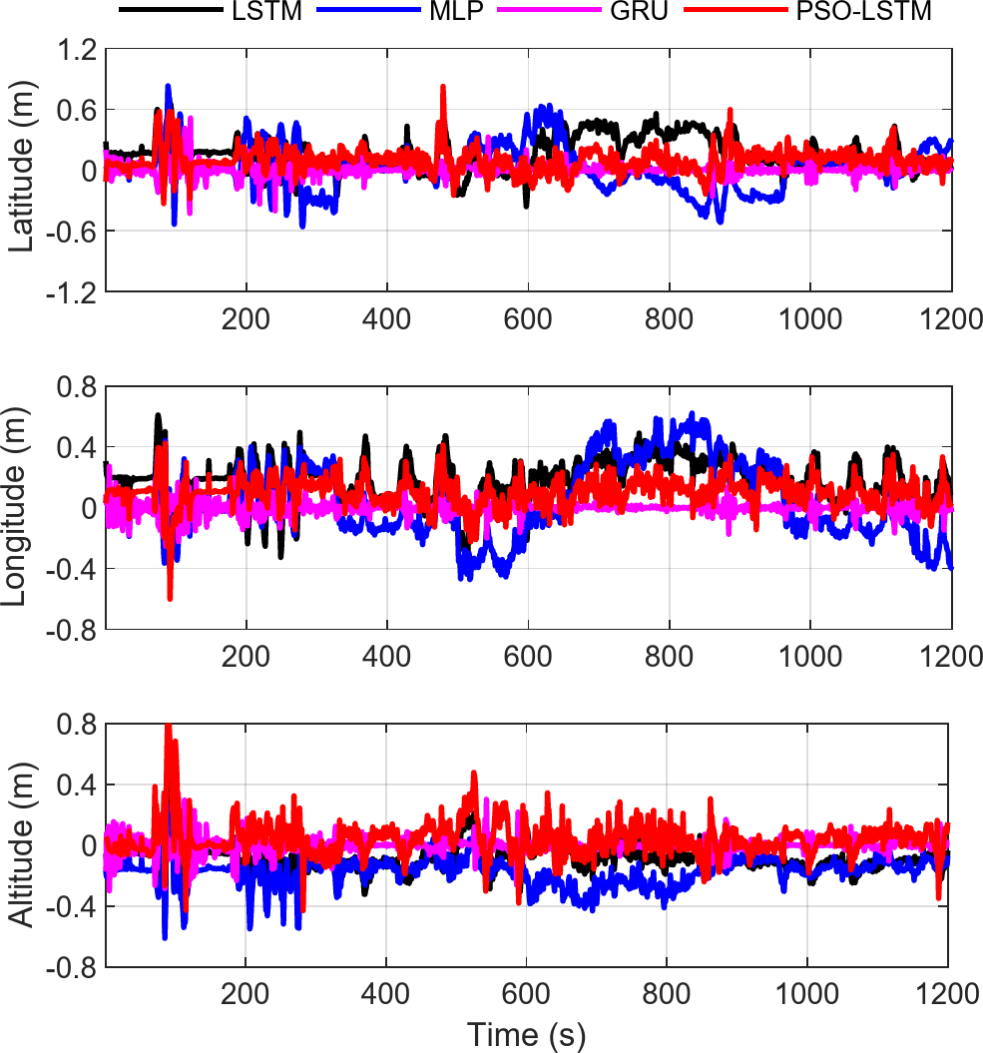
Training errors of GPS position increment in Latitude, Longitude and Altitude based on LSTM, MLP, GRU and PSO-LSTM.

**Fig. 9:**
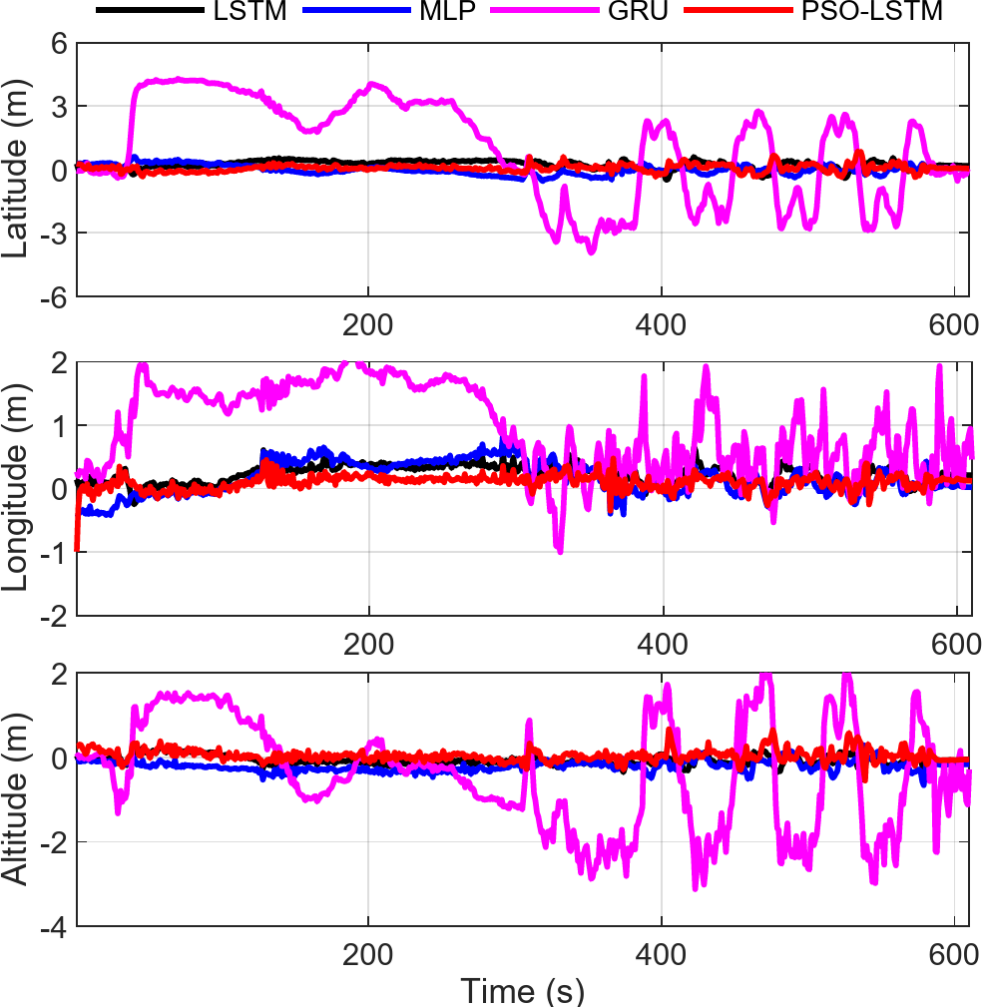
Predicting errors of GPS position increment in Latitude, Longitude and Altitude based on LSTM, MLP, GRU and PSO-LSTM.

To further validate the accuracy of vehicle displacement increment predictions using the proposed method, the specific force and angular rate outputs from the first 1200 s are used as input and the corresponding displacement increment are used as output for the training set. The input-output data from 1201 to 1810 s are used as the test set. The performance of four models LSTM, MLP, GRU, and PSO-LSTM are validated using the training and test sets. The position increment errors for the training and test sets are shown in Figs. 8 and 9. The RMS values of the increment prediction errors for both the training and testing sets in the latitude, longitude, and altitude directions are provided in Table 3. The statistical results from the figures and tables show that the position increment in all three directions has the smallest training error RMSE when using the GRU model, achieving centimeter-level accuracy. The PSO-LSTM and MLP methods perform next, with the LSTM model using empirical parameters having the largest error. The MAE statistical indicator for all four methods follows the same trend, while the differences in the R-squared values are not significant. This suggests that the R-squared values close to 0.99 indicate that all four methods are able to explain the training set data effectively, demonstrating strong model performance in capturing the underlying patterns.

The test set results indicate that the PSO-LSTM method can predict displacement increments more accurately, with the predicted RMSE error ranging between 0.154 and 0.186 m. Despite achieving the best performance on the training set, the GRU method exhibits the lowest prediction accuracy, primarily due to several factors. One possible reason is the discrepancy in the motion characteristics of the carrier between the training and test sets, which impairs the model’s ability to generalize effectively to the test set. Additionally, the GRU model may have learned noise features present in the training data, leading to overfitting.

In addition, the LSTM model using empirical parameters and the MLP model show RMSE prediction errors ranging from 0.203 to 0.285 m and 0.222 to 0.305 m, respectively. These results demonstrate that the proposed method outperforms the MLP and GRU models in both training and prediction, and also highlight that the PSO-optimized LSTM method, by finding the optimal hyperparameters, not only reduces training time but also achieves better prediction accuracy compared to the LSTM model with empirical parameters. This indicates that when GPS signals are unavailable, the proposed method effectively captures the vehicle’s motion characteristics, with the predicted displacement increments in the decimeter range. Therefore, the GPS pseudo-observations predicted by the PSO-LSTM method are selected for further anomaly detection and integrated positioning.

**Table 3:** Training and prediction errors using LSTM, MLP, GRU and PSO-LSTM model (Unit: m).

		Training error			Testing error		
		Latitude	Longitude	Altitude	Latitude	Longitude	Altitude
LSTM	RMSE	0.228	0.232	0.128	0.285	0.275	0.203
	MAE	0.181	0.203	0.109	0.246	0.239	0.114
	R-squared	0.992	0.992	0.998	0.988	0.984	0.997
MLP	RMSE	0.212	0.247	0.198	0.222	0.305	0.240
	MAE	0.080	0.093	0.082	0.258	0.254	0.262
	R-squared	0.993	0.991	0.994	0.993	0.980	0.989
GRU	RMSE	0.058	0.046	0.054	2.552	1.162	1.337
	MAE	0.034	0.028	0.031	2.220	0.983	1.112
	R^2^	0.9995	0.9997	0.9996	0.029	0.706	0.650
PSO-LSTM	RMSE	0.151	0.136	0.122	0.186	0.154	0.170
	MAE	0.119	0.116	0.083	0.140	0.126	0.107
	R-squared	0.997	0.997	0.997	0.995	0.995	0.994

### PSO-LSTM and adaptive EKF assisted positioning

When GPS signals are unavailable, relying solely on inertial sensors to estimate the vehicle’s position leads to rapid error accumulation over time. Similarly, using the proposed method to predict vehicle displacement increments based on the specific force and angular rate outputs from inertial sensors can also result in error accumulation. Therefore, calculating the position at the moment of GPS signal outages based on predicted increments cannot be directly fused with the position derived from inertial navigation. During the EKF measurement update, the adaptive factor is computed based on the predicted residual innovation and using equations (10) to (15). This approach allows for the dynamic adjustment of observation noise by leveraging historical observation information when GPS signals are available, thereby reducing or mitigating the impact of accumulated errors or gross errors on the state estimation.

**Fig. 10:**
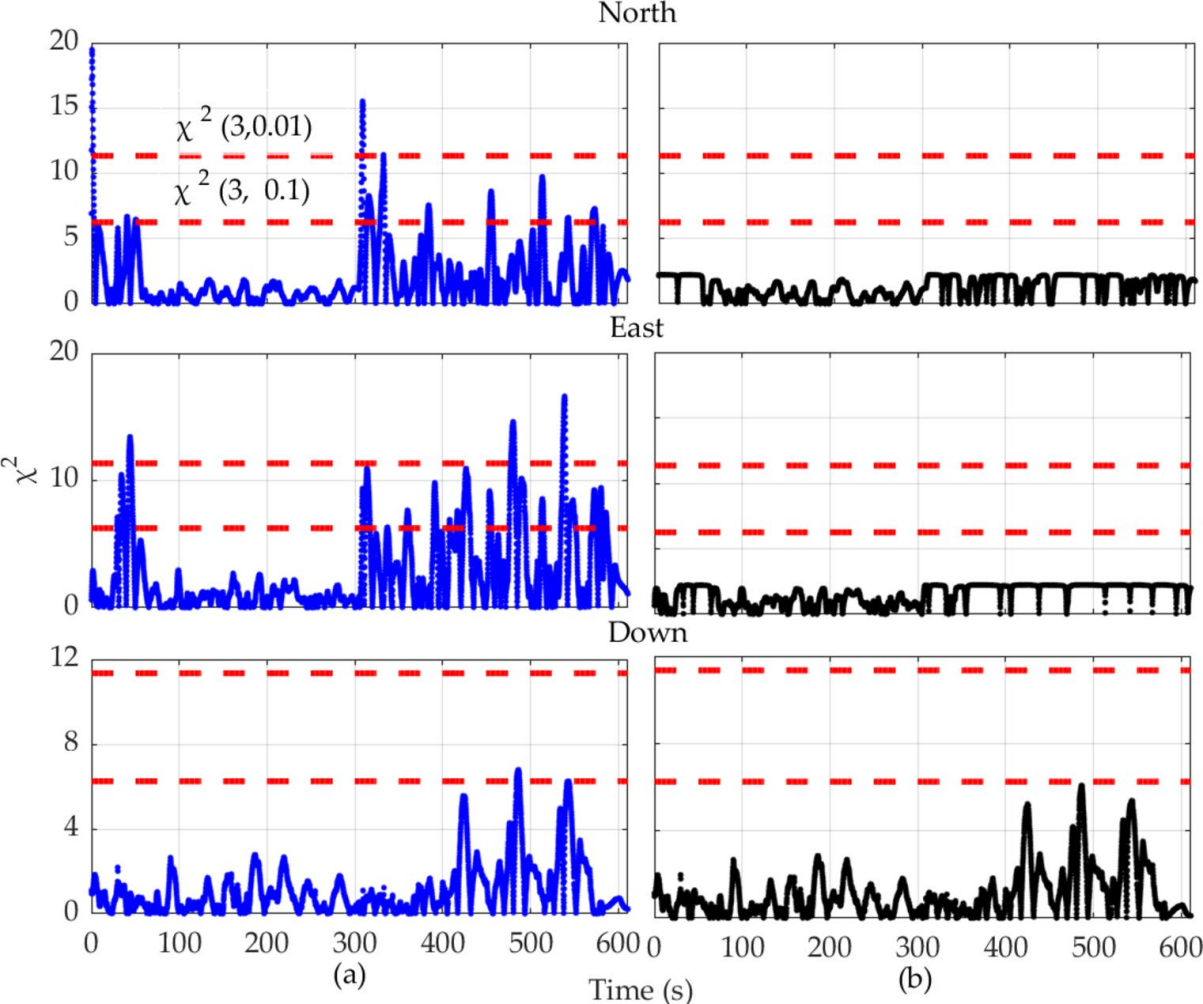
Comparison of chi-Square test results of EKF (**a**), and fading adaptive EKF (**b**), based on pseudo-observations extrapolated from PSO-LSTM.

Then, the chi-square test statistic is calculated using Eqs. (10) and (11) to assess the anomalous observation information. Figure 10 illustrates the chi-square statistics for the conventional EKF (Fig. 10 (a)) and the fading adaptive EKF (Fig. 10 (b)). It should be noted that the degrees of freedom are set to 3, with significance levels set at 0.1 and 0.01. At the 10% significance level, it is concluded that the vehicle position calculated based on the predicted displacement increments is closer to the actual vehicle position. Otherwise, the observation noise needs to be adjusted before proceeding with the filtering estimation. As shown in Fig. 9, there are indeed outliers in the vehicle positions calculated based on the predicted increments, which cannot be fully validated by the chi-square test. However, using the fading adaptive factor can further enhance the reliability of the observations.

**Fig. 11:**
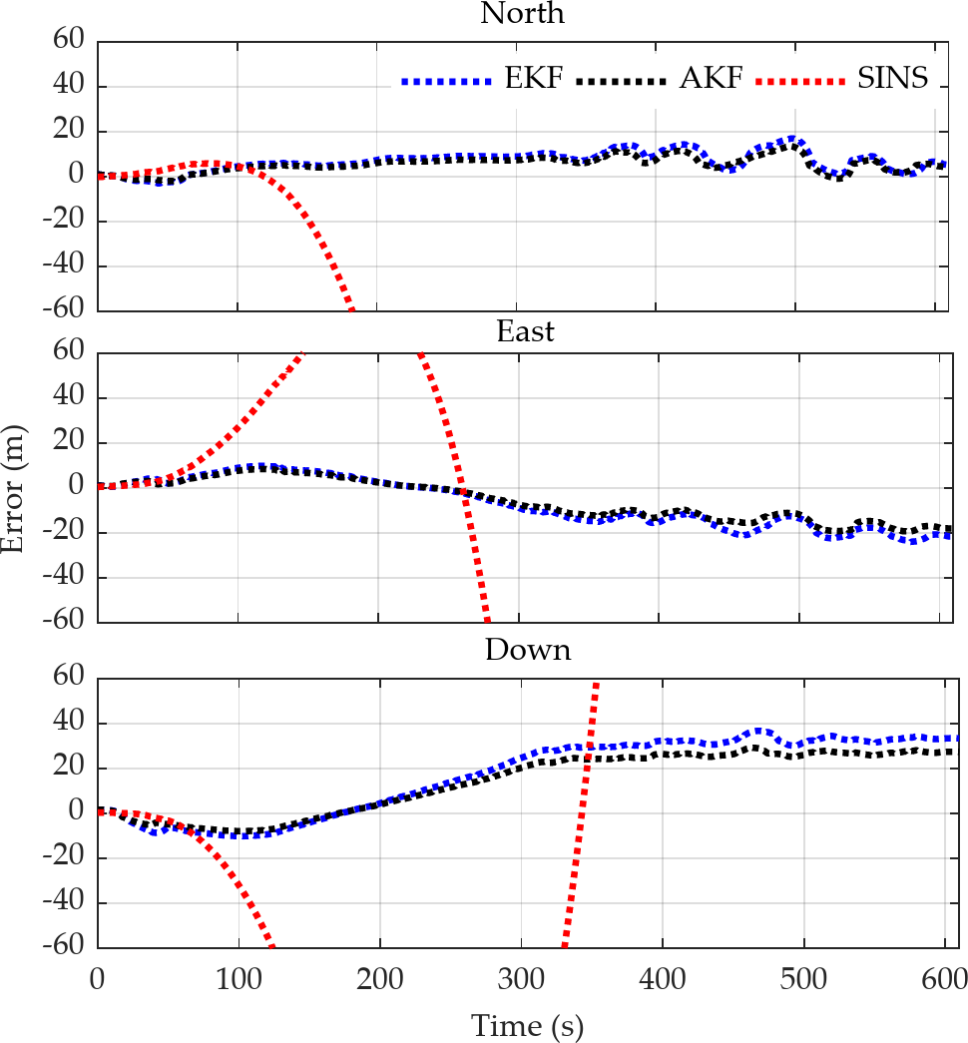
Three-Dimensional position errors of the carrier based on EKF, fading adaptive EKF, and inertial dead reckoning method.

Figure 11 shows the three-dimensional position errors of the vehicle during GPS signal unavailability, calculated using EKF, adaptive EKF (AKF), and pure inertial navigation which denoted as ‘SINS’. It can be observed from the figure that although both the inertial navigation position estimates and the vehicle pseudo-position based on predicted displacement increments exhibit error accumulation, the divergence rate of errors in the former is significantly higher than that of the latter. When GPS is unavailable for more than 100 s, the position error from inertial navigation exceeds several hundred meters, and due to the magnitude of the error, it is not fully displayed in the figure. However, the fused positioning using the predicted vehicle pseudo-position can still provide good correction for the position errors from inertial navigation. Nevertheless, as the outages time increases, the combined positioning error also shows a tendency to grow. Additionally, it can be observed from the figure that after introducing the adaptive factor, the positioning error is reduced compared to the scheme that uses only EKF. This indicates that the dynamic adjustment of observation noise mitigates the impact of observation anomalies, thereby improving positioning accuracy.

**Fig. 12:**
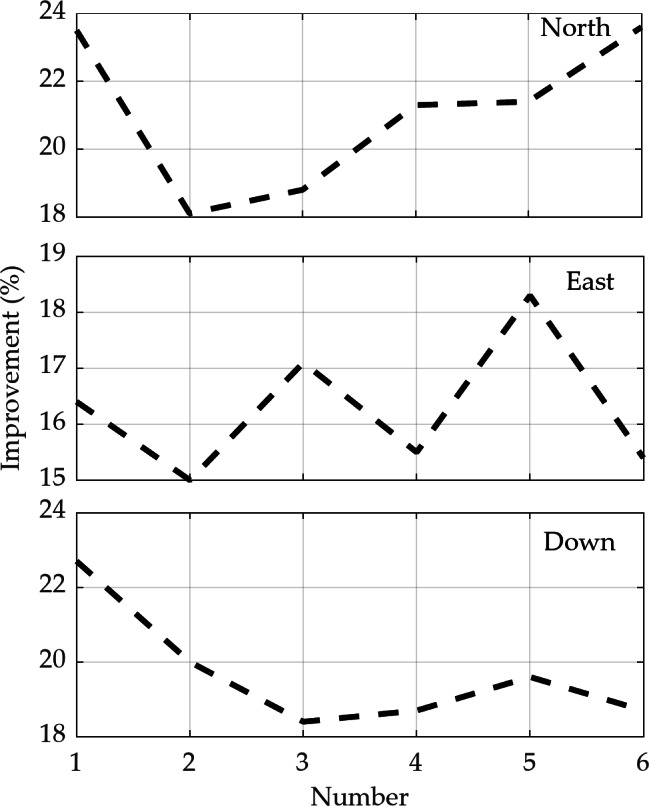
Improvement in positioning accuracy of AKF relative to EKF during GPS outages every 100 s.

To further analyze the degree of improvement in positioning accuracy of the AKF scheme compared to the EKF, the position errors of North, East, and Down directions are recorded every 100 s to calculate the RMSEs for both methods. The statistical results and percentage improvements are presented in Table 4; Fig. 12, respectively. It can be observed that as the duration of GPS unavailability increases, the positioning errors in all three directions increase to varying degrees. The positioning errors of the AKF scheme are lower than those of the EKF. Among the six statistical results, the maximum improvement in the North direction is approximately 23.6%, in the East direction is about 18.3%, and in the Down direction is around 22.7%. The average RMSEs for the positioning errors of the EKF scheme in each period are 7.163, 11.020, and 20.178 m, while the AKF scheme yields errors of 5.664, 9.227, and 16.294 m, respectively. It should be noted that the positioning error in the north direction is smaller after 500 s than the error between 400 and 500 s, which may be due to the movement of the vehicle on the circular track. The prediction accuracy is better during straight motion than during turns. Additionally, the position estimated by the SINS is corrected by the feedback correction from the EKF and AEKF estimation results. During the 100 s of movement after 500 s, the feedback correction accuracy is better than the previous results. These results further demonstrate the effectiveness of the proposed fading adaptive filtering in integrated positioning, enhancing the reliability of machine learning-based assistance for positioning during GPS unavailability.

**Table 4:** The RMSs of positioning error in North, East and down direction every 100 s (Unit, m).

Time (s)		1-100	101–200	201–300	301–400	401–500	501–600
North	EKF	2.204	5.135	8.171	9.810	11.255	6.404
	AKF	1.687	4.205	6.632	7.717	8.850	4.895
East	EKF	4.216	6.983	4.169	13.295	16.410	21.047
	AKF	3.525	5.939	3.456	11.232	13.406	17.803
Down	EKF	8.119	6.821	14.208	28.234	31.697	31.988
	AKF	6.275	5.454	11.599	22.951	25.484	25.999

## Conclusions

In GPS/INS integrated navigation positioning, when the GPS receiver is unable to receive GPS signals normally due to the influence of the motion environment, the performance of integrated navigation will significantly degrade. To address this issue, a method for auxiliary positioning based on the LSTM model for predicting vehicle displacement increments is proposed. This method uses the specific force output from the accelerometer and the angular rate output from the gyroscope as inputs to the model, with the vehicle’s displacement increments as the model output. Due to the significant impact of the number of neurons and learning rate hyperparameters in the LSTM model on its training and prediction performance, relying on empirical values does not guarantee optimal results. A particle swarm optimization algorithm is employed to find the global optimal values for these two hyperparameters.

The results indicate that after six iterations of optimization, global optimal values can be obtained for the latitude, longitude, and elevation directions. Considering that the predicted vehicle displacement increments may also experience error accumulation, which could lead to abnormal prediction residuals, a fading adaptive factor is introduced. This factor utilizes the predicted residual information and fully leverages historical observation data to dynamically adjust the observation noise covariance matrix, thereby mitigating or suppressing the impact of observation anomalies on the filtering estimation results. When the duration of GPS signal unavailability exceeds 100 s, the position error from pure inertial navigation can reach several hundred meters. However, the vehicle pseudo-position prediction method based on the PSO-LSTM model effectively corrects the position errors from pure inertial navigation, significantly suppressing error divergence. Furthermore, the proposed fading adaptive EKF shows varying degrees of improvement in positioning accuracy compared to the conventional EKF in the North, East, and Down directions, with average enhancements of approximately 21.1%, 16.3%, and 19.7%, respectively. This indicates the effectiveness and feasibility of the proposed method during periods of GPS unavailability.

## Data Availability

The datasets used and/or analysed during the current study available from the corresponding author on reasonable request.

## References

[1] Yi, Y. & On improving the accuracy and reliability of GPS/INS-based direct sensor georeferencing., PhD Thesis, The Ohio State University., (2007).

[2] Proletarsky, A. V., Neusypin, K. A. & Selezneva, M. S. Method for Improving Accuracy of INS using Scalar Parametric Identification, in *International Russian Automation Conference (RusAutoCon)*, Sochi, Russia: IEEE, Sep. 2019, pp. 1–4. (2019). 10.1109/RUSAUTOCON.2019.8867706

[3] Neusypin, K., Kupriyanov, A., Maslennikov, A. & Selezneva, M. Investigation into the nonlinear Kalman filter to correct the INS/GNSS integrated navigation system. *GPS Solut.* **27** (2), 91. 10.1007/s10291-023-01433-5 (2023).

[4] Shang, X., Sun, F., Zhang, L., Cui, J. & Zhang, Y. Detection and mitigation of GNSS spoofing via the pseudorange difference between epochs in a multicorrelator receiver. *GPS Solut.* **26** (2), 37. 10.1007/s10291-022-01224-4 (2022).

[5] Li, H., Borhani-Darian, P., Wu, P. & Closas, P. Deep neural network correlators for GNSS multipath mitigation. *IEEE Trans. Aerosp. Electron. Syst.* 1–23. 10.1109/TAES.2022.3197098 (2022).

[6] Tian, Y., Liu, Z., Lin, M. & Li, K. Modelling and mitigation of GNSS multipath effects by least-squares collocation considering Spatial autocorrelation. *J. Geod.* **97** (4), 37. 10.1007/s00190-023-01726-0 (Apr. 2023).

[7] Abosekeen, A., Iqbal, U., Noureldin, A. & Korenberg, M. J. A Novel Multi-Level Integrated Navigation System for Challenging GNSS Environments, *IEEE Trans. Intell. Transport. Syst.* **22** (8) 4838–4852. 10.1109/TITS.2020.2980307 (Aug. 2021).

[8] Wang, S. et al. A Bioinspired Navigation System for Multirotor UAV by Integrating Polarization Compass/Magnetometer/INS/GNSS, *IEEE Trans. Ind. Electron.* **70** (8) 8526–8536. 10.1109/TIE.2022.3212421 (Aug. 2023).

[9] Zhang, Z., Niu, X., Tang, H., Chen, Q. & Zhang, T. GNSS/INS/ODO/wheel angle integrated navigation algorithm for an all-wheel steering robot. *Meas. Sci. Technol.* **32** (11), 115122. 10.1088/1361-6501/ac17fb (2021).

[10] Zhou, T., Hasheminasab, S. M. & Habib, A. Tightly-coupled camera/lidar integration for point cloud generation from GNSS/INS-assisted UAV mapping systems. *ISPRS J. Photogrammetry Remote Sens.* **180**, 336–356. 10.1016/j.isprsjprs.2021.08.020 (2021).

[11] Qian, C. et al. An integrated GNSS/INS/LiDAR-SLAM positioning method for highly accurate forest stem mapping. *Remote Sens.* **9** (1) 3. 10.3390/rs9010003 (Dec. 2016).

[12] Gray, R. A. & Maybeck, P. S. An integrated GPS/INS/baro and radar altimeter system for aircraft precision approach landings, in *Proceedings of the IEEE 1995 National Aerospace and Electronics Conference. NAECON 1995*, Dayton, OH, USA: IEEE, pp. 161–168. (1995). 10.1109/NAECON.1995.521930

[13] Tal, A., Klein, I. & Katz, R. Inertial navigation system/doppler velocity log (INS/DVL) fusion with partial DVL measurements. *Sensors* **17** (2), 415. 10.3390/s17020415 (2017). 28241410 10.3390/s17020415 PMC5335996

[14] Fernandes, M. R., Magalhães, G. M., Zúñiga, Y. R. C., Do, J. B. R. & Val GNSS/MEMS-INS integration for drone navigation using EKF on lie groups. *IEEE Trans. Aerosp. Electron. Syst.* **59** (6), 7395–7408. 10.1109/TAES.2023.3290575 (2023).

[15] Yang, Y., Wang, X., Zhang, N., Gao, Z. & Li, Y. Artificial neural network based on strong track and square root UKF for INS/GNSS intelligence integrated system during GPS outage. *Sci. Rep.* **14** (1), 13905. 10.1038/s41598-024-64918-4 (2024). 38886514 10.1038/s41598-024-64918-4 PMC11183257

[16] Liu, J., Cai, B., Tang, T. & Wang, J. A CKF based GNSS/INS train integrated positioning method, in *IEEE International Conference on Mechatronics and Automation*, Xi’an, China: IEEE, Aug. 2010, pp. 1686–1689. (2010). 10.1109/ICMA.2010.5588839

[17] Wang, J., Chen, X. & Shi, C. A novel robust iterated CKF for GNSS/SINS integrated navigation applications. *EURASIP J. Adv. Signal. Process.* **2023**, 83. 10.1186/s13634-023-01044-9 (2023).

[18] Fang, Y., He, X., Xu, Y., Luo, Q. & Rui, K. A New GNSS/INS Navigation Scheme Using Integrated Particle Filter and Extended Kalman Filter, in *IEEE International Conference on Smart Internet of Things (SmartIoT)*, Xining, China: IEEE, Aug. 2023, pp. 16–22. 10.1109/SmartIoT58732.2023.00012 (2023).

[19] Hu, G., Gao, S., Zhong, Y., Gao, B. & Subic, A. Modified federated Kalman filter for INS/GNSS/CNS integration, *Proceedings of the Institution of Mechanical Engineers, Part G: Journal of Aerospace Engineering* **230** (1) 30–44 10.1177/0954410015586860 (2016).

[20] Han, H., Xu, T., Li, R., Ma, W. & Wu, H. An improved multiple-outlier-robust GNSS/INS EKF filer based on multiple statistical similarity measure. *Meas. Sci. Technol.* **35** (12), 126308. 10.1088/1361-6501/ad78f7 (2024).

[21] Yang, Y. & Cui, X. Adaptively robust filter with multi adaptive factors, *Survey Review* **40** (309) 260–270. (Jul. 2008). 10.1179/003962608X325330

[22] Jiang, C., Zhang, S., Li, H. & Li, Z. Performance evaluation of the filters with adaptive factor and fading factor for GNSS/INS integrated systems. *GPS Solut.* **25** (4), 130. 10.1007/s10291-021-01165-4 (2021).

[23] Cheng, S. et al. Adaptive non-holonomic constraint aiding Multi-GNSS PPP/INS tightly coupled navigation in the urban environment. *GPS Solut.* **27** (3), 152. 10.1007/s10291-023-01475-9 (2023).

[24] Li, B. & Chen, G. Improving the combined GNSS/INS positioning by using tightly integrated RTK. *GPS Solut.* **26** (4), 144. 10.1007/s10291-022-01331-2 (2022).

[25] Gao, W., Zhan, X. & Yang, R. INS-aiding information error modeling in GNSS/INS ultra-tight integration. *GPS Solut.* **28** (1), 35. 10.1007/s10291-023-01574-7 (2024).

[26] Ebrahimi, A., Nezhadshahbodaghi, M., Mosavi, M. R. & Ayatollahi, A. An improved GPS/INS integration based on EKF and AI during GPS outages. *J. Circuit Syst. Comp.* **33** (02), 2450035. 10.1142/S021812662450035X (2024).

[27] Zhao, S., Zhou, Y. & Huang, T. A novel method for AI-Assisted INS/GNSS navigation system based on CNN-GRU and CKF during GNSS outage. *Remote Sens.* **14** (18), 4494. 10.3390/rs14184494 (2022).

[28] Tang, Y. et al. A GRU and AKF-Based hybrid algorithm for improving INS/GNSS navigation accuracy during GNSS outage. *Remote Sens.* **14** (3), 752. 10.3390/rs14030752 (2022).

[29] Xu, Y. et al. Motion-Constrained GNSS/INS integrated navigation method based on BP neural network. *Remote Sens.* **15** (1). 10.3390/rs15010154 (2022).

[30] Yuan, Y., Wang, Y., Gao, W. & Shen, F. Vehicular relative positioning with measurement outliers and GNSS outages. *IEEE Sens. J.* **23** (8), 8556–8567. 10.1109/JSEN.2023.3250617 (2023).

[31] Zhang, T. et al. INS-Aided GNSS Pseudo-Range error prediction using machine learning for urban vehicle navigation. *IEEE Sens. J.* **24** (6), 9135–9147. 10.1109/JSEN.2024.3355705 (2024).

[32] Tan, X., Wang, J., Jin, S. & Meng, X. GA-SVR and Pseudo-position-aided GPS/INS integration during GPS outage. *J. Navig.* **68** (4), 678–696 (2015).

[33] Liu, X., Wang, W., Guo, Z., Wang, C. & Tu, C. Research on adaptive SVR indoor location based on GA optimization. *Wirel. Pers. Commun.* **109** (2), 1095–1120. 10.1007/s11277-019-06605-6 (2019).

[34] Hasanipanah, M., Shahnazar, A., Bakhshandeh Amnieh, H. & Jahed Armaghani, D. Prediction of air-overpressure caused by mine blasting using a new hybrid PSO–SVR model. *Eng. Comput.* **33** (1), 23–31. 10.1007/s00366-016-0453-2 (2017).

[35] Zhang, B., Zhao, W., Zou, S., Zhang, H. & Luan, Z. A reliable vehicle lateral velocity Estimation methodology based on SBI-LSTM during GPS-Outage. *IEEE Sens. J.* **21** (14), 15485–15495. 10.1109/JSEN.2020.3022056 (2021).

[36] Dhake, H., Kashyap, Y. & Kosmopoulos, P. Algorithms for Hyperparameter Tuning of LSTMs for Time Series Forecasting, *Remote Sensing*. 15(8) (2076). 10.3390/rs15082076 (2023).

[37] Fang, W. et al. A LSTM algorithm estimating Pseudo measurements for aiding INS during GNSS signal outages. *Remote Sens.* **12** (2). 10.3390/rs12020256 (2020).

[38] Taghizadeh, S. & Safabakhsh, R. An integrated INS/GNSS system with an attention-based hierarchical LSTM during GNSS outage. *GPS Solut.* **27** (2), 71. 10.1007/s10291-023-01412-w (2023).

[39] Titterton, D. & Weston, J. Strapdown inertial navigation technology. *IEEE Aerosp. Electron. Syst. Mag*. **20** (7), 33–34. 10.1109/MAES.2005.1499250 (2005).

[40] Parsopoulos, K. E. & Vrahatis, M. N. Recent approaches to global optimization problems through particle swarm optimization. *Nat. Comput.* **1** (2/3), 235–306. 10.1023/A:1016568309421 (2002).

